# High-risk subtype: Clinical manifestations and molecular characteristics of submandibular gland adenoid cystic carcinoma

**DOI:** 10.3389/fonc.2022.1021169

**Published:** 2022-12-16

**Authors:** Mengjiao Zhou, Tingyao Ma, Xuelian Wang, Shujing Zhang, Guoliang Yang, Ruohui Song, Xiaohong Chen

**Affiliations:** ^1^ Department of Otolaryngology, Head and Neck Surgery, Beijing Tongren Hospital, Capital Medical University, Key Laboratory of Otolaryngology Head and Neck Surgery, Ministry of Education, Capital Medical University, Beijing, China; ^2^ Department of Otolaryngology, the First Affiliated Hospital of Anhui University of Chinese Medicine, Hefei, Anhui, China

**Keywords:** adenoid cystic carcinoma, lung metastasis, MYB, parotid gland, submandibular gland

## Abstract

**Objective:**

Adenoid cystic carcinoma of the head and neck mainly occurs in the major salivary glands, of which the parotid gland and submandibular gland are the most common. The purpose of this study was to clarify the site-specific differences in prognosis and molecular expression characteristics of the patients and to achieve stratified risk management of the clinical prognosis.

**Materials:**

By performing a single-centre retrospective analysis combined with analyses of the Surveillance, Epidemiology, and End Results (SEER) database, cBioPortal and GEO databases, the clinical prognostic characteristics and the differences in molecular expression patterns of ACC in the submandibular gland and parotid gland were analysed. Cox regression analysis, the chi-square test, Fisher’s test and the log-rank test were used to compare the significance of differences.

**Results:**

Compared with patients with parotid gland ACC, the submandibular gland ACC is more likely to have metastases in the cervical lymph node (21.7% vs. 3.3%) and shows a higher rate of distant metastasis within 1 year after the primary site diagnosis (47.8% vs. 23.3%), a worse overall prognosis, more frequent mutations of *MYB/MYBL1* (50% vs. 25%) and abnormal upregulation of the phosphatidylinositol-3 kinase (PI3K) pathway.

**Conclusions:**

Submandibular gland ACC is associated with site-specific early cervical lymph node metastasis and hidden distant metastasis, along with rapid progression and a poor prognosis. A high *MYB/MYBL1* mutation rate and abnormal upregulation of the PI3K pathway with MYB involvement were identified.

## 1 Introduction

Adenoid cystic carcinoma (ACC), a rare malignant tumor originating from secretory glands, occurs most commonly in the head and neck. The main clinical features of ACC are relentless and slow growth, perineural invasion, and a high distant metastasis rate. ACC is composed of the epithelial and myoepithelial cells, which are arranged into three pathological subtypes: cribriform, tubular and solid (most of which are mixed). Therefore, the pathological manifestation of ACC is biphasic differentiation. Surgery combined with radiotherapy is the conventional treatment for the primary tumor. Distant metastasis developed in 52% of patients, mainly within the first 5 years following diagnosis, and the median time to metastasis was only 31.5 months (1). The most common site of distant metastasis is the lung, accounting for 67-85.9% of cases, followed by the bone and liver (2, 3). The 5-year overall survival rate(OS) is 68%, and once metastasis occurs, the median survival time is only 20-32 months (4). The special pathological subtypes (solid or high-grade transformation), T/N stages, and treatment of the primary sites are common clinical risk factors for ACC lung metastasis (5–8). The high incidence rate and uncontrollable continuous progression of distant metastasis are challenges in the treatment of this disease.

Achieving population stratification by screening high-risk factors and adopting different clinical intervention measures are very important. Renata Ferrarotto (2021) analysed two ACC molecular subtypes defined by MYC and P63 using RNA sequencing (RNA-seq) and revers-phase protein microarray (RPPA). The high-risk type of ACC-I showed substantial overexpression of MYC and MYC target genes, mRNA splicing, and enrichment for NOTCH-activating mutations, resulting in shorter median survival than patients with ACC-II (3.44 years vs. 23.2 years) (9). In addition to molecular typing, clinical characteristics might also identify high-risk groups for simple and effective risk stratification and management.

The parotid gland and submandibular gland are the most common salivary glands in the head and neck (64.8%) (10). The salivary glands site was a prognostic risk factor in the nomogram model developed by Xiaoli Mu (11), but the specific classification of salivary glands was not performed. Jason Tasoulas et al. found that the overall prognosis of patients with submandibular gland ACC in stage IV was worse than that of patients with ACC in the parotid gland and minor salivary glands (10), but the specific differences in the clinical prognosis presentation and molecular expression patterns were not clarified. We further confirmed whether the prognosis of patients with submandibular gland ACC and parotid gland ACC differed in a large multicentre sample, and analysed the reasons for the difference. Therefore, based on the retrospective case data from our hospital combined with the SEER, cBioPortal and GEO data, we analysed the specific clinical manifestations and molecular characteristics of submandibular gland ACC by comparing it with parotid gland ACC which has a similar gene expression background and acinar structure with submandibular gland (12).

## 2 Materials and methods

### 2.1 Data sources

#### 2.1.1 SEER database

**Figure 1:**
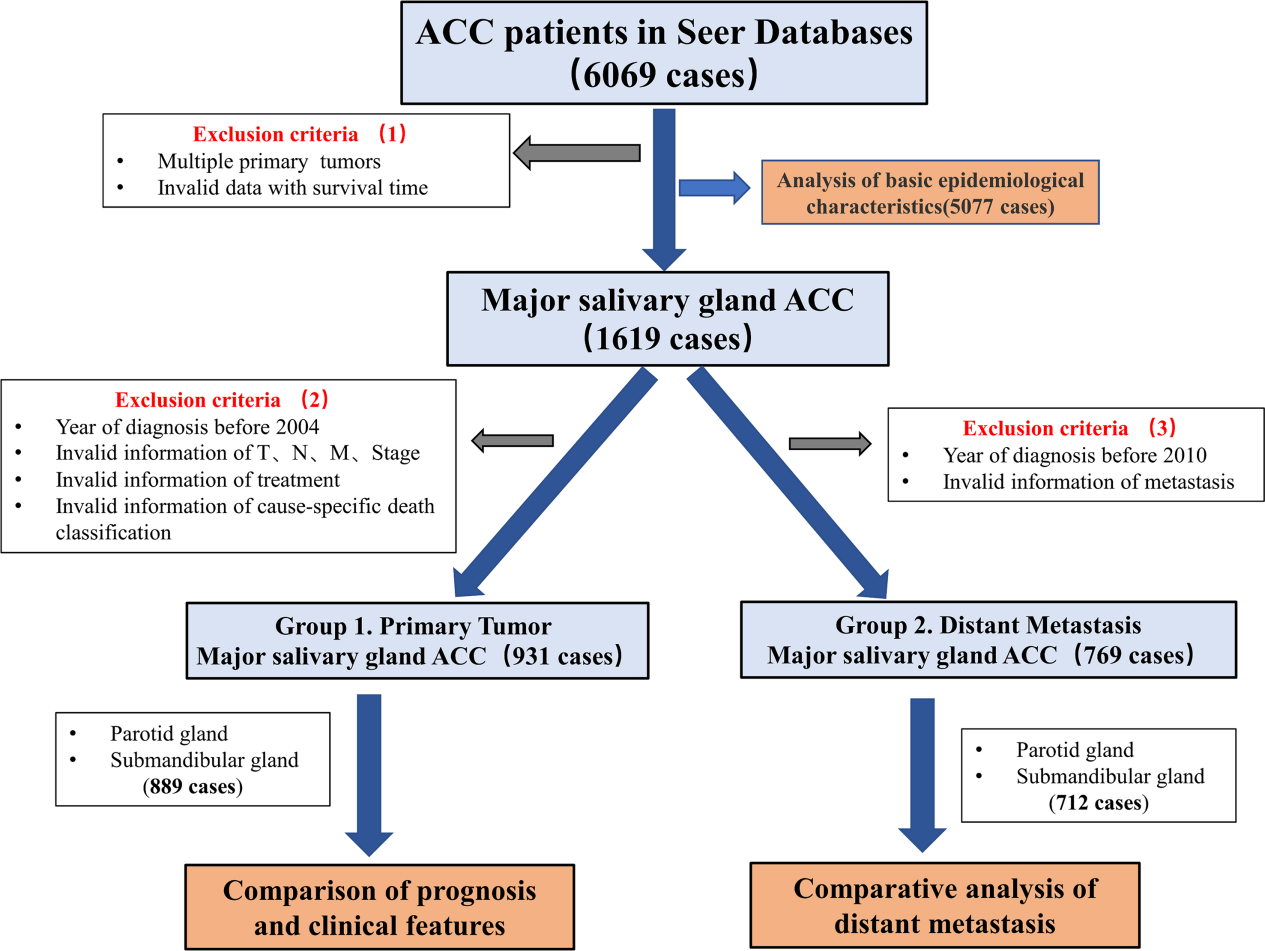
Flow chart showing the screening process for data from the SEER database. The dataset **“**Incidence-SEER Research Plus Data, 18 Registries, Nov 2020 Sub (2000-2018)**”**, including a total of 6069 patients with ACC, was selected based on screening criteria 1. The basic epidemiological characteristics of 5077 ACC patients with complete information were analysed. Then, a total of 1619 patients with major salivary glands ACC were selected. According to screening criteria 2, patients with missing basic clinical characteristics were excluded from the 1619 patients for the analysis of the differences in clinical characteristics to determine the prognosis. According to screening criteria 3, 769 patients were selected from the original 1619 patients, and distant metastasis was analysed.

The dataset “Incidence-SEER Research Plus Data, 18 Registries, Nov 2020 Sub (2000-2018)” was downloaded from the SEER database using SEER Stat 8.4.0 software. Case screening criteria were based on the study by Jason Tasoulas et al. (10), as follows:

1. The screened tumor type was ACC in the classification of salivary gland tumors determined by the World Health Organization (WHO). The SEER database code was based on the International Classification of Cancer Diseases, the third edition (ICD-O-3) system (ACC = 8200), and patients with ACC were initially screened.
2. The main specific information included age, sex, race, primary site, TNM stage, surgery (primary site and cervical lymph node), radiotherapy, chemotherapy, SEER cause-specific death classification, survival in months, and extent of disease—SEER Combined Mets to DX-lung/liver/bone/brain.
3. As TNM staging was not available in the data before 2004, data collected after 2004 were selected. Cases from 2004 to 2009 were graded according to the American Joint Committee on Cancer (AJCC) 6th edition, while cases from 2010 and later were graded according to the AJCC 7th edition. The staging criteria for major salivary gland tumors were not significantly revised in the 7th edition; cases classified according to the 6th and 7th editions of the AJCC were merged. Invalid cases, which have multiple primary tumors, ambiguous survival time, or non-specific death classification, were removed. (**Figure 1**).

#### 2.1.2 Collection of clinical data from patients

A retrospective analysis was performed on patients (including outpatients and inpatients) who were diagnosed with head and neck ACC in Beijing Tongren Hospital from January 2005 to March 2022, and the primary sites were the parotid gland and submandibular gland. The basic information was complete. Clinical and pathological characteristics, such as sex, symptoms at first diagnosis, TNM stage (based on the eighth edition of AJCC), treatment method, pathological grade, perineural invasion, and the Ki67 index, were collected. The pathological grading criteria used by Szanto et al. (13) were uniformly modified to grade I-II and grade III. Pulmonary metastases were diagnosed by two or more lung CT reports (at intervals of 3 months or more) with the persistent progression of pulmonary nodules and after the exclusion of other diseases, and the diagnosis of extrapulmonary metastasis was made by performing a PET-CT evaluation.

#### 2.1.3 Mutation and RNA-seq analyses

Data on mutations in parotid and submandibular glands ACC, including the mutation frequency and mutation type, were obtained from the cBioPortal database (https://www.cbioportal.org/). ACC was searched and the following datasets were selected: (J Clin Invest 2019), (Fmi. Am J Surg Pathl.2014), (JHU, Cancer Prev Res 2016), (MDA. Clin Cancer Res 2015), (MGH. Nat Gen 2016), (MSKCC. NAT Genet 2013), and (Sanger/Mda.jCI 2013). The information collected included the site, age, histological type, and perineural invasion. The histological type was uniformly modified into grade I-II and grade III according to (13) the pathological grading criteria. The mutational landscape was mapped using Microsoft Office Home and Student 2019. The parotid gland and submandibular gland were selected as the “Tumor Disease Anatomic Site”. “Cancer Gene” was screened as defined by OncoKB from mutated genes/structural variant genes, and the number of mutations in each sample should be greater than 2.

The GSE88804 and GSE34816 datasets were screened in the GEO database and corrected for batch effects, and the parts were selected as parotid gland and submandibular gland ACC for the differential expression analysis. The differential expression analysis was performed using R 4.2.0 and the R limma package (|log fold change| > 0.585, P value < 0.05). Differentially expressed genes were selected for a Kyoto Encyclopedia of Genes and Genomes (KEGG) enrichment analysis using the HIPLOT enrichment database (public/db/kegg/hsa_kegg_20220424.rds) (https://hiplot.com.cn/). Bubble charts were drawn using the web tool Sangerbox3.0 (http://vip.sangerbox.com/home.html). Gene set enrichment analysis (GSEA) was performed using a web tool (http://www.webgestalt.org/). Venn diagrams were constructed and analysed using the web tool (https://hiplot.com.cn/basic/).

#### 2.1.4 Statistical analysis

Cox regression analysis, the chi-square test, Fisher’s test, and the log-rank test were performed on the data using SPSS 22.0 and GraphPad Prism 8.0 software. Survival curves were drawn using the Kaplan-Meier method. P<0.05 (*), P<0.01 (**), and P<0.001 (***) indicate a significant difference. The web tool Sangerbox3.0 was used to draw the forest map (http://vip.sangerbox.com/home.html).

## 3 Results

### 3.1 Epidemiological characteristics and prognosis of patients with submandibular gland ACC based on the SEER database

**Figure 2:**
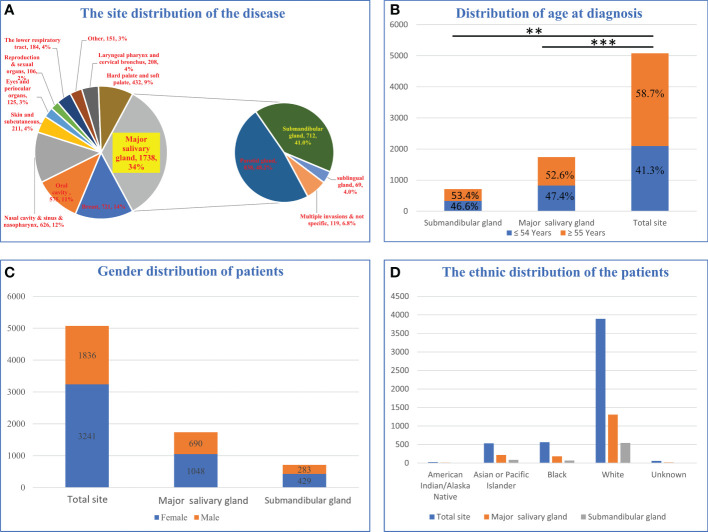
Epidemiological analysis of patients with submandibular gland ACC. **(A)** Distribution of disease sites in 5077 patients. **(B)** Age distribution of submandibular gland ACC compared with the major salivary glands and all parts of the body. **(C)** Sex distribution of patients with submandibular gland ACC compared with major salivary glands and body parts. **(D)** The racial distribution of submandibular gland ACC compared with major salivary glands and body parts (** P-value <0.01; *** P-value <0.001).

After downloading the dataset and performing the initial screening (**Figure 1**, Exclusion criteria 1), a total of 5077 ACC patients were screened, among which the patients with ACC of the major salivary glands accounted for 34%. The submandibular gland (41.0%, 712/1738) and parotid gland (48.2%, 838/1438) were the most common sites of tumors in the major salivary glands (**Figure 2A**). The age group with the highest proportion of ACC in the submandibular gland was 40-54 years old, and the incidence of ACC in the submandibular gland was higher in younger patients (P<0.05, **Figure 2B**, **Table S1**). The incidence rate of ACC in the major salivary glands and submandibular gland in females was approximately 1.5 times that in males, and white people were the most frequently affected (**Figures 2C, D**).

Two groups of data were obtained after screening based on two criteria (**Figure 1**). Group 1 was used to compare the clinical characteristics of 931 patients with primary site tumors after removing 42 patients with incomplete data. A total of 889 patients were obtained, including 477 patients with parotid gland ACC and 412 patients with submandibular gland ACC. The median follow-up times were 93 months and 100 months, respectively (the mean follow-up times were 95 months and 100 months, respectively).

**Figure 3:**
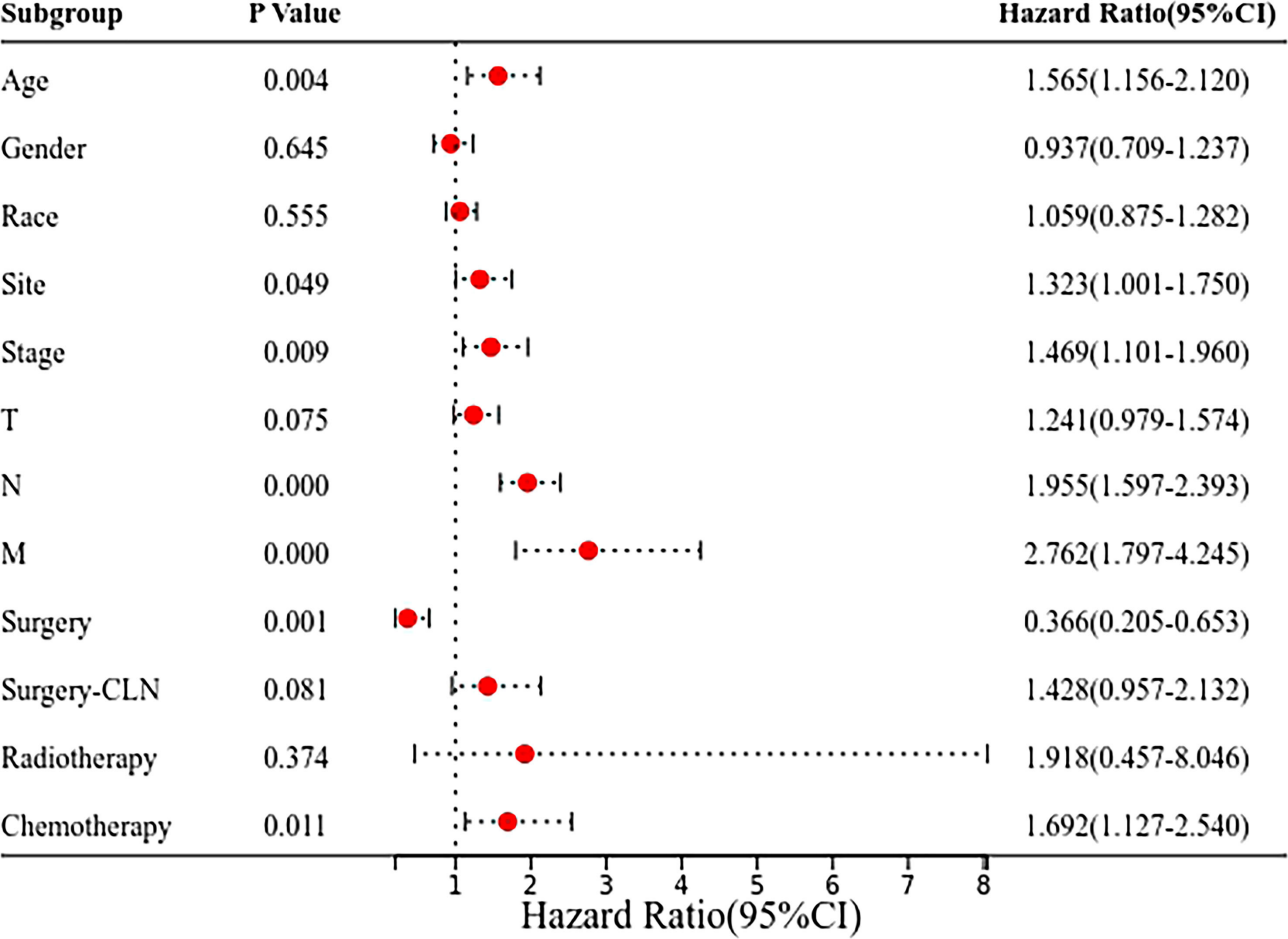
Forest plot of the Cox regression analysis of 899 patients with submandibular/parotid glands ACC in the SEER database. The clinical prognosis of 899 patients with parotid gland and submandibular gland ACC was determined using the Cox regression model to analyse the factors influencing the prognosis, such as the tumor site.

**Figure 4:**
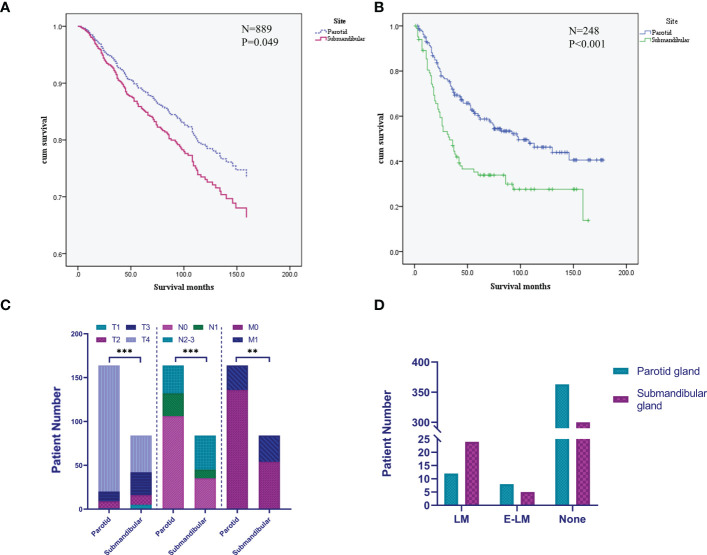
Differences in the prognosis and clinical characteristics of patients with submandibular/parotid gland ACC in the SEER database. **(A)** Survival curves for patients with submandibular and parotid glands ACC analysed using the Cox regression model. **(B)** Survival curves for patients with stage IV submandibular and parotid glands ACC analysed using the Kaplan-Meier method. **(C)** Difference in the distribution of TNM staging in patients with stage IV submandibular and parotid glands ACC. **(D)** Distribution of distant metastases of submandibular and parotid glands ACC. LM, lung metastasis; E-LM, extrapulmonary metastasis; None, no distant metastasis found (** P-value <0.01; *** P-value <0.001).

The Cox regression analysis showed that age, site, T stage, N stage, M stage and chemotherapy were the related factors affecting the prognosis (P<0.05), and the overall prognosis of patients with submandibular gland ACC was poor (P<0.05) (**Figures 3** and **4A**). Basic clinical features were analysed and the log-rank test revealed that age, tumor stage, T stage, N stage, M stage, and surgical treatment at the primary site correlated with the prognosis (P<0.05, **Table S2**). However, differences in the prognosis of patients with parotid gland ACC and submandibular gland ACC were significant only in patients with stage IV tumors (P<0.001, **Figure 4B**), consistent with the results reported by Jason Tasoulas et al. (10). Further analysis of the distribution characteristics of the stage IV population showed that compared with patients with parotid gland ACC, the proportion of patients with stage T4 submandibular gland ACC was lower (50% vs. 87.8%), while the rates of lymph node metastasis (58.3% vs. 35.4%) and distant metastasis (35.7% vs. 17.1%) were higher (P<0.01, **Figure 4C**). Compared with patients with parotid gland ACC, patients with submandibular gland ACC showed mainly stage I-III tumors (79.6% vs. 65.6%) and T1-3 tumors (89.8% vs. 69.8%) (**Table 1**, P<0.05). Distant metastasis was evaluated in Group 2 (post-2010 dataset), including 383 patients with parotid gland ACC (mean follow-up time: 49 months, median follow-up time: 44 months) and 329 patients with submandibular gland ACC (mean follow-up time: 51 months, median follow-up time: 50 months). The overall distant metastasis rate of patients with submandibular gland ACC was higher than that of patients with parotid gland ACC (8.81% vs. 5.22%, **Figure 4D**), and the lung metastasis rate was higher (7% vs. 3%). In conclusion, compared with parotid gland ACC, submandibular gland ACC has a worse prognosis in general, with a higher rate of distant metastasis, mainly lung metastasis, at an early stage (within 1 year).

**Table 1:** Differences in the clinical characteristics of patients with submandibular and parotid glands ACC from the SEER database.

		Major salivary gland (2004-2018)		X2
		Parotid	Submandibular	P value
**Age**	<50	182 (38.2%)	143 (34.7%)	0.287
	≥50	295 (61.8%)	269 (65.3%)	
**Gender**	Male	186 (39.0%)	152 (36.9%)	0.52
	Female	291 (61%)	260 (63.1%)	
**Race**	White	362 (75.9%)	308 (74.8%)	0.116
	Black	57 (11.9%)	35 (8.5%)	
	Other	54 (11.3%)	65 (15.8%)	
	NA	4 (0.8%)	4 (1%)	
**Stage**	I	126 (26.4%)	126 (30.6%)	**<0.001**
	II	104 (21.8%)	110 (26.7%)	
	III	83 (17.4%)	92 (22.3%)	
	IV	164 (34.4%)	84 (20.4%)	
**T**	T1	136 (28.5%)	134 (32.5%)	**<0.001**
	T2	117 (24.5%)	128 (31.1%)	
	T3	80 (16.8%)	108 (26.2%)	
	T4	144 (30.2%)	42 (10.2%)	
**N**	N0	399 (83.6%)	342 (83.0%)	0.196
	N1	46 (9.6%)	31 (7.5%)	
	N2-N3	32 (6.7%)	39 (9.5%)	
**M**	M0	449 (94.1%)	382 (92.7%)	0.395
	M1	28 (5.9%)	30 (7.3%)	
**Surgery**	None	33 (6.9%)	18 (4.4%)	0.103
	Yes	444 (93.1%)	394 (95.6%)	
**Cervical lymph node dissection**	None	141 (29.6%)	177 (43.0%)	**<0.001**
	Yes	336 (70.4%)	235 (57.0%)	
**Radiotherapy**	None/NA	7 (1.5%)	6 (1.5%)	0.989
	Yes	470 (98.5%)	406 (98.5%)	
**Chemotherapy**	None/NA	435 (91.2%)	376 (91.3%)	0.972
	Yes	42 (8.8%)	36 (8.7%)	
**Total**		477	412	

### 3.2 Clinicopathological features and manifestations of lung metastases of ACC of the submandibular gland (retrospective analysis of patients from a single centre)

Seventy-six patients with ACC (parotid gland ACC (30) and submandibular gland ACC (46)) were included in our analysis. The mean follow-up time was 66 months (median 55 months) for patients with parotid gland ACC and 58 months (median 47 months) for patients with submandibular gland ACC. Compared with patients with parotid gland ACC, more patients with submandibular gland ACC were older than 50 years of age (45.7%, 21/46) and had a higher rate of cervical lymph node metastasis (21.7% vs. 3.3%). No significant differences were observed in the pathological grade, neurotropic growth, Ki67 index or treatment methods (including general treatment, radiotherapy and neck dissection) (**Table 2**).

**Table 2:** Differences in the overall clinical characteristics of patients with submandibular and parotid glands ACC based on a single**-**centre retrospective analysis.

		Major salivary gland (Stage IV)		X2
		Parotid	Submandibular	P value
**Age**	<50	23 (76.7%)	25 (54.3%)	**0.049**
	≥50	7 (23.3%)	21 (45.7%)	
**Gender**	Male	12 (40%)	15 (32.6%)	0.51
	Female	18 (60%)	31 (67.4%)	
**Symptom**	Painless mass	17 (56.7%)	37 (80.4%)	**0.026**
	Pain and other discomfort	13 (43.3%)	9 (19.6%)	
**Histological grade**	NA	4 (13.3%)	4 (8.7%)	0.839
	Grade I—II	16 (53.3%)	25 (54.3%)	
	Grade III	10 (33.3%)	17 (37.0%)	
**Perineural invasion**	NA	6 (20.0%)	9 (19.6%)	0.752
	None	5 (16.7%)	5 (10.9%)	
	Yes	19 (63.3%)	32 (69.6%)	
**Ki67**	NA	1 (3.3%)	10 (21.7%)	0.127
	<30%	19 (63.3%)	23 (50.0%)	
	30%-60%	9 (30.0%)	11 (23.9%)	
	>60%	1 (3.3%)	2 (4.3%)	
**T**	T1	3 (10.0%)	8 (17.4%)	0.317
	T2	14 (46.7%)	24 (52.2%)	
	T3	5 (16.7%)	9 (19.6%)	
	T4	8 (26.7%)	5 (10.9%)	
**N**	N0	29 (96.7%)	36 (78.3%)	**0.042**
	N+	1 (3.3%)	10 (21.7%)	
**M**	M0	27 (90.0%)	34 (73.9%)	0.085
	M1	3 (10.0%)	12 (26.1%)	
**Treatment**	S	3 (10%)	5 (10.9%)	0.1
	S+R	24 (80.0%)	26 (56.5%)	
	S+R+C	3 (10.0%)	10 (21.7%)	
	S+C	0 (0.0%)	5 (10.9%)	
**Cervical lymph node dissection**	None	20 (66.7%)	27 (58.7%)	0.484
	Yes	10 (33.3%)	19 (41.3%)	
**Radiotherapy**	None	3 (10.0%)	10 (21.7%)	0.256
	<60GY	6 (20.0%)	12 (26.1%)	
	≥60GY	21 (70.0%)	24 (52.2%)	
**Total**		30	46	

S, surgery; R, radiotherapy; C, chemotherapy.

**Figure 5:**
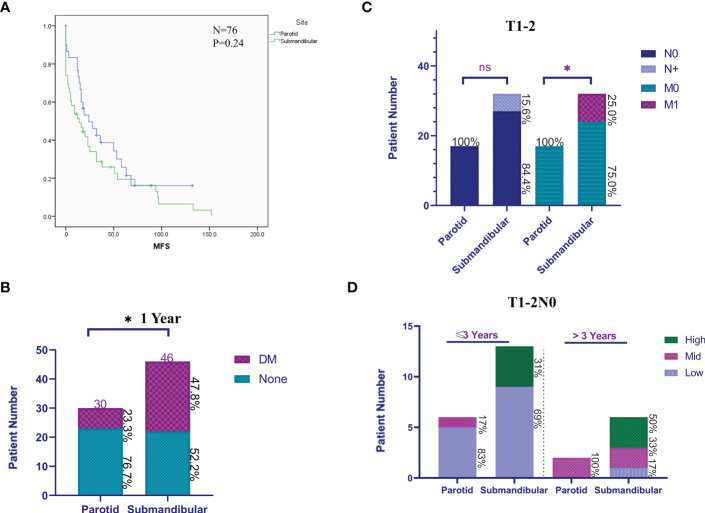
Clinical features of patients with submandibular/parotid glands ACC from a single**-**centre retrospective analysis. **(A)** The Kaplan-Meier method was used to analyse the differences in distant metastases from ACC of the submandibular and parotid glands. **(B)** The difference in distant metastases (DM) within 1 year after the primary diagnosis of submandibular and parotid glands ACC. **(C)** Cervical lymph node and distant metastasis ratio at the first diagnosis of early (T1-2) submandibular and parotid glands ACC. **(D)** Early (T1-2N0) differences in the rate of progression of distant metastases of submandibular and parotid glands ACC. High: prognostic high-risk status for distant metastasis; Mid: prognostic medium-risk status for distant metastasis; and Low: prognostic low-risk status for distant metastasis (ns: P-value> 0.05; * P-value <0.05).

When comparing distant metastases at the first diagnosis and follow-up, patients with submandibular gland ACC had a mean distant metastasis-free survival (MFS) of 32 months (median 14 months), while those with parotid gland ACC had a mean value of 44 months (median 24 months); however, the log-rank test did not reveal a significant difference (P>0.05, **Figure 5A**). The rate of distant metastasis in patients with submandibular gland ACC (47.8%) was higher than that in patients with parotid gland ACC (23.3%) within 1 year after the primary diagnosis (P<0.05, **Figure 5B**). Compared with parotid gland ACC and submandibular gland ACC in the early stage (T1-2), the rate of distant metastasis of submandibular gland ACC was significantly higher at the first diagnosis (P<0.05), and the rate of cervical lymph node metastasis (N+) was slightly higher (**Figure 5C**).

We subsequently screened and analysed patients with complete information on distant metastasis identified at the first diagnosis of primary early-stage (T1-2N0) ACC, including 8 patients with parotid gland ACC (62%, 8/17) and 19 patients with submandibular gland ACC (73%, 19/27) to compare the difference in disease status at the time of the first diagnosis of distant metastasis. We identified a significant difference in the risk level of the disease course of the first diagnosis of distant metastasis. The risk classification is as follows: low-risk, multiple nodules in both lungs and the largest diameter is <1 cm, without extrapulmonary metastasis; medium-risk, multiple nodules in both lungs and the largest is 1-3 cm in diameter, without extrapulmonary metastasis; and high-risk, multiple nodules in both lungs with a maximum diameter greater than 3 cm or with extrapulmonary metastasis. The analysis of the distribution of risk grades of distant metastasis in patients with parotid gland ACC and submandibular gland ACC within 3 years and after 3 years showed that the high-risk grade of distant metastasis in patients with submandibular gland ACC was 31% and 50%, respectively, while the high-risk grade of distant metastasis in patients with parotid gland ACC was 0 (**Figure 5D**). In conclusion, patients with ACC of the submandibular gland are prone to early occult distant metastasis, and the disease progresses rapidly.

### 3.3 Mutation map and expression characteristics of ACC oncogene in submandibular gland

**Figure 6:**
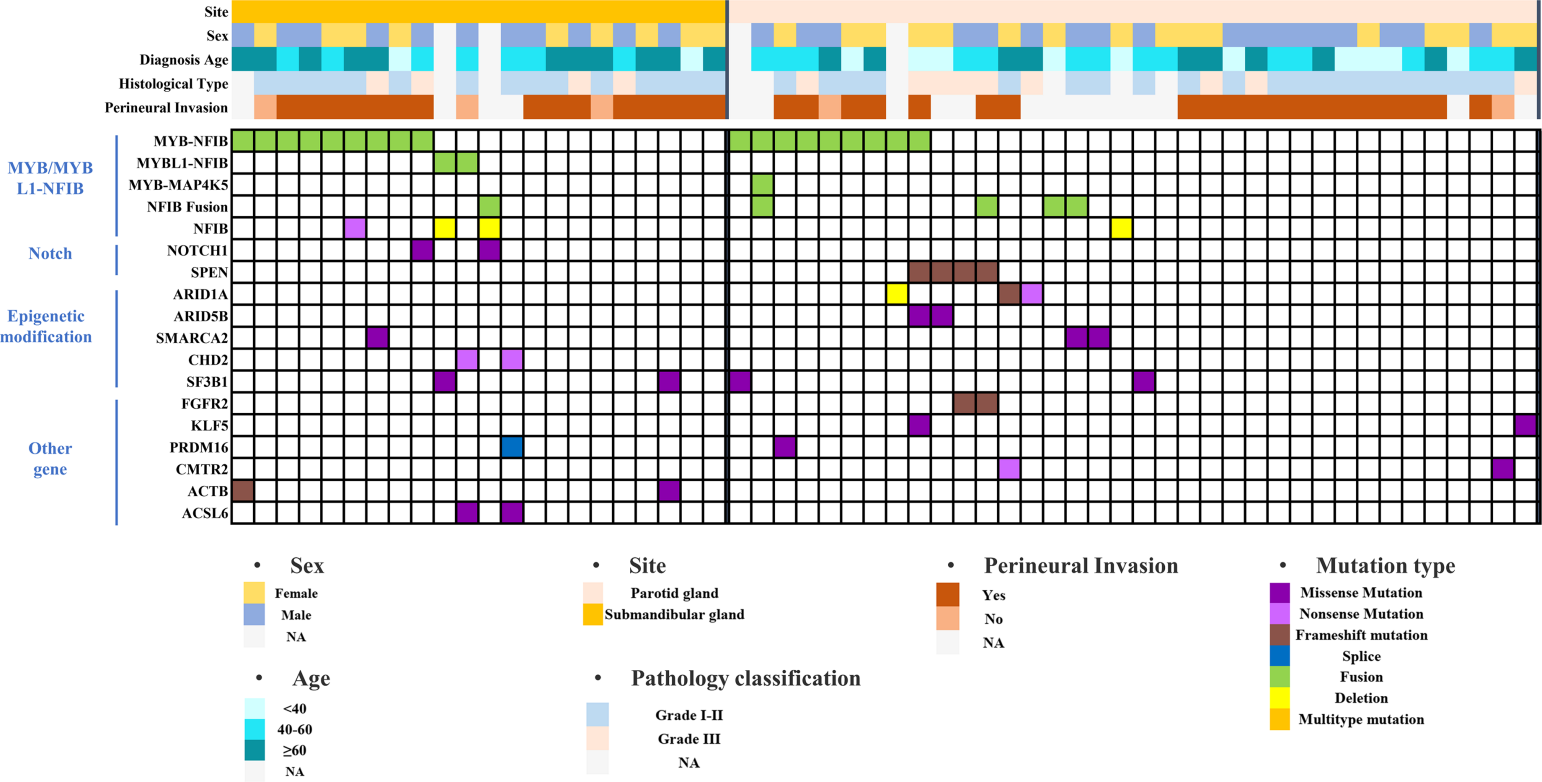
Mutation landscape of submandibular gland and parotid gland ACC based on the cBioPortal database.

The dataset was searched using the cBioPortal database, and 22 cases of submandibular gland ACC and 36 cases of parotid gland ACC were screened for gene mutation analysis (**Figure 6**). The mutant genes were divided into four categories: *MYB/MYBL1-NFIB*-related fusion genes, Notch pathway-related genes, epigenetic modification-related genes, and others. The *MYB/MYBL1-NFIB* fusion had a higher mutation frequency in submandibular gland ACC compared with submandibular gland ACC (50% vs. 25%), and the fusion mutation of *MYBL1* only appeared in submandibular gland ACC (9%). *NOTCH1* mutation did not occur in ACC of the parotid gland, accounting for 9% of ACC of the submandibular gland; meanwhile, mutations in *SPEN* (a negative regulator of the Notch pathway) did not appear in ACC of the submandibular gland, and the mutation frequency in ACC of the parotid gland was 11%. Epigenetic modification-related genes included *ARID1A*, *ARID5B*, *SMARCA2*, *CHD2*, and *SF3B1*. Except for *CHD2* (9%), all of them were detected in parotid and submandibular gland ACC, and all of them had mutually exclusive mutations.

**Figure 7:**
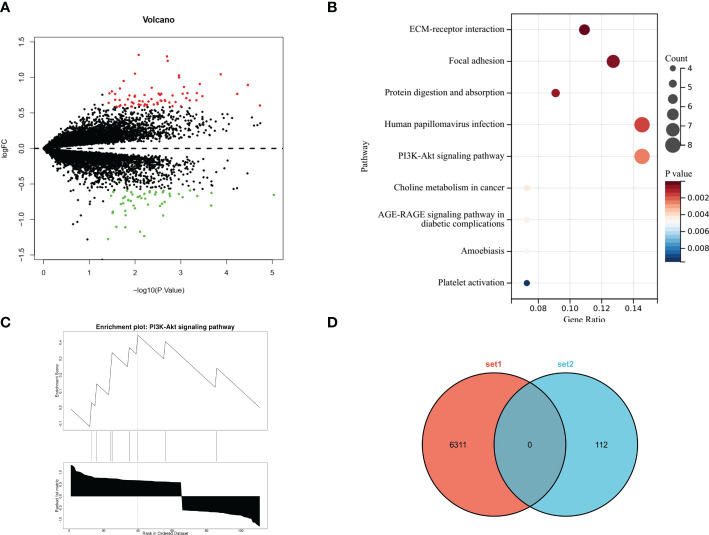
RNA sequencing results from the submandibular gland and parotid gland ACC based on the GEO database. **(A)** Volcano plot showing differentially expressed genes. Red dots and green dots indicate upregulated and downregulated genes, respectively, in submandibular gland ACC compared with parotid gland ACC. **(B)** Bubble diagram of the KEGG enrichment analysis showing the results for differentially expressed genes. The colour and size of bubbles correspond to the P value and the number of enriched genes, respectively. **(C)** GSEA of differentially expressed genes revealed that the PI3K signaling pathway was upregulated in ACC of the submandibular gland. **(D)** Venn diagram of gene set intersections: set 1 is the differentially expressed genes in the normal parotid gland and submandibular gland tissues, and set 2 is the differentially expressed genes in the parotid gland and submandibular gland ACC tissues.

We further studied the differences in the molecular expression patterns of ACC tumor tissues in the parotid gland and submandibular gland and analysed the abnormal pathways in submandibular gland ACC by screening upregulated and downregulated genes in 10 cases of submandibular gland ACC and 16 cases of parotid gland ACC from the two GEO datasets. *MYB* gene expression was significantly upregulated in submandibular gland ACC compared with parotid gland ACC (**Figure 7A**). KEGG enrichment analysis of genes with P<0.05 and |log FC|>0.585 showed that genes were significantly enriched in the PI3K pathway, including the *PDGFA, MYB, ITGA2, FN1, EGF, COL6A3, COL1A2*, and *COL1A1* genes (**Figure 7B**). According to GSEA, the activity of the PI3K pathway was upregulated (**Figure 7C**). Next, we aimed to exclude differences in the expression of genes related to the PI3K pathway in normal parotid and submandibular glands tissues, and 6311 differentially expressed genes (P<0.05, |log FC|>1) in the normal parotid gland and submandibular gland tissues were downloaded from the supplementary materials of the study by Marie Saitou et al. (12) and intersected with the differentially expressed genes in the submandibular gland and parotid gland ACC obtained from the GEO dataset. No intersecting genes were identified (**Figure 7D**). This result excluded the possibility that differentially expressed genes in tumor genes were caused by differences in the genetic background of normal tissues. Therefore, based on the results from the cBioPortal and GEO databases, submandibular gland ACC has a higher frequency of *MYB/MYBL1* mutations, and genes in the PI3K pathway, including MYB, are upregulated.

## 4 Discussion

ACC is a rare malignant tumor of glandular origin that may occur systemically but is more common in the head and neck. The development of uncontrolled distant metastases after primary surgery has become a major challenge in disease treatment. Risk stratification based on differences in prognosis is a prerequisite for individualized treatment of the disease. Renata Ferrarotto proposed in 2017 that patients with *NOTCH1* mutations should be defined as a population with a poor prognosis who are prone to have extrapulmonary metastasis (14); In 2021, they proposed two risk subtypes defined by MYC and TP63: ACC-I (37%) and ACC-II (63%). High-risk ACC-I is characterized by the enrichment of NOTCH-activating mutations and overexpression of MYC target genes, and mRNA splicing. The continuous improvement of molecular typing is helpful for the precise treatment of diseases, and risk stratification based on clear clinical features can simply and effectively assist with clinical individual treatment. Moreover, molecular typing should be further defined and improved based on molecular expression characteristics in populations with varying clinical prognostic performance.

Identifying the site-specific clinical prognosis of patients with submandibular gland ACC is helpful for simple and effective clinical treatment stratification and an accurate risk assessment. In the nomogram prediction model constructed by Ian Ganly, tumors in the nasal cavity and paranasal sinuses had the highest risk, followed by the major salivary glands, while tumors in the larynx/pharynx/oral cavity had the lowest risk. However, none of the studies further analysed the prognostic risk weights for the three major salivary glands (15, 16). A log-rank univariate analysis performed by Jason Tasoulas using SEER data revealed that the prognosis of patients with submandibular gland ACC was worse than that of patients with parotid gland and minor salivary glands ACC which was only showed in stage IV. But the specific manifestation of the clinical prognosis difference has not been compared and expounded in detail (10). We screened 5077 patients with ACC from the SEER database, a large sample clinical database. Using Cox regression analysis, patients with submandibular gland ACC were found to have a worse prognosis than patients with parotid gland ACC, consistent with the research conclusions described above. Because normal tissues of the parotid and submandibular glands have similar gene expression patterns (12), the submandibular gland ACC can be compared with the parotid gland ACC to determine the specific clinical prognosis and abnormal molecular expression. Similar to the study by Jason Tasoulas et al, the same analysis showed that the prognosis of patients with stage IV submandibular gland ACC was significantly worse than the patients with parotid gland ACC (10). Further analysis and comparison indicated that ACC of the parotid gland was characterized by a higher T stage, while ACC of the submandibular gland was characterized by a higher rate of cervical lymph node metastasis and distant metastasis. Although the log-rank test showed that a higher TNM stage and T stage are high-risk factors, the finding that the proportion of stage IV and T4 stage parotid gland ACC was higher than that in the submandibular gland ACC seems paradoxical, as the parotid gland has a larger space for invasion inside and outside the envelope and is prone to a later T stage. Subsequently, a dataset with complete distant metastasis information was selected for further analysis, and submandibular gland ACC had a higher overall metastasis rate than parotid gland ACC, and the lung was the main site of metastasis. In conclusion, compared with parotid gland ACC, submandibular gland ACC has a higher metastasis rate and worse prognosis.

In addition, the aforementioned study based on the SEER database found that the submandibular ACC was more likely to have lymph node metastasis and earlier distant metastasis than the parotid gland in stage IV. Because clinical staging adopts a mixed grading method compared with TNM staging, it cannot reflect the inherent law of tumor occurrence and development. Moreover, the SEER database lacks the specific time and disease progression status of distant metastasis. Therefore, we expanded the sample size as much as possible in a single**-**centre retrospective cohort to analyse the specific characteristics of distant metastases in submandibular gland ACC and to analyse the difference in the progression rate of lymph node and distant metastases of submandibular gland ACC compared with parotid gland ACC. Limited by the heterogeneity and the small size of the population, we did not observe a significant difference in the distant metastasis time of the two sites of ACC tumors using the log-rank test; however, within 1 year after diagnosis, submandibular gland ACC had a higher rate of distant metastasis (P<0.05). At the same time, in patients with T1-2 stage, submandibular gland ACC had a higher rate of cervical lymph node metastasis and distant metastasis than parotid gland ACC at the first diagnosis (P<0.05). Further comparison of the metastatic status of patients with T1-2N0 stage parotid gland ACC and submandibular gland ACC showed that patients with submandibular gland ACC had a higher risk level of distant metastasis (pulmonary metastatic nodules >3 cm or extrapulmonary metastasis). In conclusion, compared with patients with parotid gland ACC, patients with submandibular gland ACC have a risk of early occult metastasis and rapid disease progression, resulting in a poor site-specific prognosis.

To further explore the molecular expression characteristics of ACC in the submandibular gland with high metastasis and poor prognosis, we analyzed the gene mutation characteristics based on the cBioPortal dataset and found that there was a high *MYB/MYBL1-NFIB* fusion ratio in the submandibular gland. Differentially expressed genes were analysed using the GEO database to further verify the differences in expression and abnormal pathways, and we found that the MYB-dominated PI3K pathway was also significantly enriched and upregulated in the submandibular gland ACC. However, the upregulation of genes in the PI3K pathway, including MYB, in the submandibular gland ACC was not due to a difference in expression between normal tissues of the parotid and submandibular glands (**Figure 7D**). *MYB* fusion mutations (the most common fusion *NFIB*) are hallmark molecular events in the development of ACC, with a mutation frequency of 16-100% (17–19), and high expression of MYB is closely related to the poor prognosis (20). Relevant studies have found that the genes of the PI3K pathway amplified and mutated in various tumors, such as breast cancer and gastric cancer, and this signaling pathway plays a role in cell survival, angiogenesis, lymph node metastasis, and distant metastasis (21, 22). In salivary gland cancers, aggressive tumor types have higher genomic alterations in the PI3K pathway (23). Therefore, the upregulation of the PI3K pathway may be the main reason for the higher rate of lymph node and distant metastasis of submandibular gland ACC than parotid gland ACC. One study found that the administration of PI3K inhibitors to the ACC xenograft mouse model effectively reduces the primary tumor burden and lung metastasis (24). Target therapies against abnormal genetic alterations have shown varying degrees of promise in the clinic as precision medicines of ACC.

## Data availability statement

The original contributions presented in the study are included in the article/**Supplementary Material**. Further inquiries can be directed to the corresponding authors.

## Author contributions

Conception and design: MZ and TM. Data analysis: XW, SZ, and GY. Manuscript writing: MZ and TM. Manuscript revision: RS and XC. Funding support: XC. All authors contributed to the article and approved the submitted version.

## Supplementary material

Supplementary Table 1 Analysis of the age distribution of patients with submandibular gland ACC in the SEER database.

Supplementary Table 2 Overall clinical characteristics of 889 patients with submandibular/parotid glands ACC in the SEER database.
